# Pore-scale investigation of the use of reactive nanoparticles for in situ remediation of contaminated groundwater source

**DOI:** 10.1073/pnas.1918683117

**Published:** 2020-06-02

**Authors:** Tannaz Pak, Luiz Fernando de Lima Luz, Tiziana Tosco, Gabriel Schubert Ruiz Costa, Paola Rodrigues Rangel Rosa, Nathaly Lopes Archilha

**Affiliations:** ^a^Engineering Department, Teesside University, Middlesbrough TS1 3BX, United Kingdom;; ^b^Chemical Engineering Department, Federal University of Parana, Curitiba 81531-980, Brazil;; ^c^Department of Environment, Land and Infrastructure Engineering, Politecnico di Torino, Torino 10129, Italy;; ^d^Brazilian Synchrotron Light Laboratory, Brazilian Center for Research in Energy and Materials, 13083-970 Campinas, São Paulo, Brazil

**Keywords:** NAPL in situ degradation, groundwater remediation, nanoremediation, zero-valent iron nanoparticle, X-ray–computed microtomography

## Abstract

Nanoscale zero-valent iron (nZVI) particles have excellent capacity for in situ remediation of groundwater resources contaminated by a range of organic and inorganic contaminants. Chlorinated solvents are by far the most treated compounds. Studies at column, pilot, and field scales have reported successful decrease in contaminant concentration upon injection of nZVI suspensions in the contaminated zones. However, the field application is far from optimized, particularly for treatments at—or close to—the source, in the presence of residual nonaqueous liquid (NAPL). The knowledge gaps surrounding the processes that occur within the pores of the sediments hosting those contaminants at microscale limit our ability to design nanoremediation processes that are optimized at larger scales. This contribution provides a pore-scale picture of the nanoremediation process. Our results reveal how the distribution of the trapped contaminant evolves as a result of contaminant degradation and generation of gaseous products. We have used state-of-the-art four-dimensional (4D) imaging (time-resolved three-dimensional [3D]) experiments to understand the details of this degradation reaction at the micrometer scale. This contribution shows that the gas released (from the reduction reaction) remobilizes the trapped contaminant by overcoming the capillary forces. Our results show that the secondary sources of NAPL contaminations can be effectively treated by nZVI, not only by in situ degradation, but also through pore-scale remobilization (induced by the evolved gas phase). The produced gas reduces the water relative permeability to less than 1% and, therefore, significantly limits the extent of plume migration in the short term.

## Significance

Chlorinated solvents are among the most recalcitrant aquifer contaminants, which can cause serious health problems such as kidney and liver damage, and some are considered carcinogenic. They are a global problem due to their wide industrial use since the beginning of the 20th century (e.g., in metal-processing plants). Conventionally, pump and treat technology (ex situ method) has been used to treat such contaminated groundwater resources. Recently, in situ techniques have been applied to lower the remediation costs (e.g., energy/water consumption) while also limiting the disruption. Nanoremediation is a new in situ technology that has shown promising results at laboratory, pilot, and field scales. This study uses 4D (time-resolved 3D) imaging to capture the dynamics of nanoremediation at the pore scale.

**Fig. 1.**
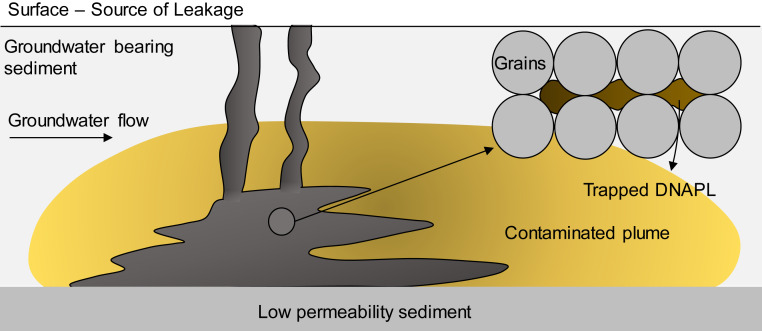
An aquifer contaminated by DNAPL showing the contamination source (gray) and plume (yellow). At the pore scale, the DNAPL phase is trapped in the form of disconnected ganglia (brown).

The release of chlorinated solvents in groundwater resources is a widespread and a global problem (1). This family of contaminants is poorly soluble and denser than water (dense nonaqueous liquid [DNAPL] phase) and, hence, can sink into deeper sediments located below a leakage source, resulting in a residual saturation of the DNAPL phase, which slowly releases the contaminants and may impact groundwater for decades. The natural flow of the groundwater forms a contaminated plume downstream of the DNAPL source (Fig. 1). Moreover, DNAPLs often have a strong affinity with natural organic matter (NOM) present in the subsoil and tend to adsorb on it. As a consequence, DNAPLs are typically present in three phases: as a residual saturation, dissolved in water, and adsorbed on the soil matrix, with the first phase acting as a long-term source for the other two. An effective remediation technology should therefore address the residual DNAPL, even if most approaches typically target the dissolved fraction.

At the pore scale, chlorinated solvents are trapped within pores of the aquifer (2, 3). Such pore-scale fluid distribution is very similar to that found in hydrocarbon reservoirs containing oil/gas (4–6) in the presence of water. The arrangement of fluids within the pore system is ruled by a range of fluid/fluid and porous media properties including interfacial tension (IFT) (7), wettability (8), and the pore-space structure (9). Wettability indicates the preference of a fluid to be in contact with a solid surface in presence of another fluid (10). Within the context of groundwater systems, chlorinated solvents are mostly nonwetting toward the host sediments.

Chlorinated hydrocarbons display limited solubility in water (in the order of grams per liter or lower). However, the legal threshold limit on the amount of these substances allowed in public water (e.g., under the Drinking Water Directive in the European Union and the Safe Drinking Water Act in the United States) is several orders of magnitude smaller than their solubility. For instance, the maximum contaminant level is 10 µg/L for trichloroethylene (TCE) in the European Union and is lower for groundwater in some countries (e.g., 1.5 µg/L in Italy). Therefore, small quantities of these DNAPLs can render large volumes of water unsafe for drinking for a long period of time. In some cases, it is possible to remove most of the DNAPL phase from the host aquifer by combining injection of water (with chemical additives) and extraction of contaminated washing solution. This process is known as aggressive soil flushing, which is similar to waterflooding in the oil industry. Such a removal of the trapped nonwetting phase is, however, always less than 100% efficient (11) at the pore scale, meaning that a portion of the DNAPL will remain trapped in the porous media (capillary trapping), forming the so-called residual DNAPL, typically in the order of a few percent of the pore-space volume. Capillary trapping is governed by the competition between the capillary and viscous forces, measured by capillary number, *N_c_* = *µV*/*σ*, where *µ* is viscosity (pascals per second), V is velocity (meters per second), and *σ* is IFT (newtons per meter).

Previous studies have shown such a trapped nonwetting phase forms droplets (occupying one pore) or larger ganglia (occupying several pores) (4, 5, 12) acting as a long-term secondary source of contamination. The secondary source of contamination is referred to as contaminated areas where the NAPL phase is sorbed and/or is present as a free phase, at residual saturation. Therefore, the NAPL phase is not mobile as an individual phase, but slowly releases the contaminant in pore water.

At the field scale, soil flushing has been shown to be poorly effective in some circumstances. In particular, where the contaminated zone is deep, controlling the flow pathways of injected/extracted water becomes very challenging. Also, heterogeneous deposits containing clay and silt lenses present challenges for soil flushing, mainly limiting the technique to the most permeable portions of the contaminated subsoil. Other disadvantages of this technology include continuous operation, high electricity consumption, and on-site treatment of extracted water. Similarly, elimination of DNAPLs through excavations followed by ex situ incineration is an unsustainable, disruptive, costly, and potentially risky (toxic by-products) process (13). Preferred technologies allow in situ contaminant degradation. Among those, bioremediation is widely practicable and cost-effective (14). Bioremediation uses microorganisms to degrade chlorinated solvents via the anaerobic processes. Although proved successful, bioremediation is limited by slow degradation rates and process complexities and is effective only for a low to moderate concentration of contaminants (i.e., typically tens of milligrams per liter) (15).

Nanoremediation is an emerging in situ remediation technology which injects nanoparticles in the form of aqueous suspensions into contaminated aquifer systems (mainly below the water table). In most applications zero-valent iron nanoparticles (nZVI) are used (16–20) even though other nanosized materials have been applied, e.g., iron oxides, graphene oxides, and carbon-based materials (21–23). nZVI is a highly reactive and excellent electron donor (Fe^0^ → Fe^2+^+ 2e^−^). Chlorinated solvents can readily accept those electrons and release their chlorine atoms in the form of ions. An example reaction is 2C_2_HCl_3_ + 3Fe^0^ + 6H^+^→2C_2_H_4_ + 6Cl¯+ 3Fe^2+^. The produced hydrocarbon is a gas. The reaction consumes hydrogen ions which are supplied either by the aquifer water or by the carrier aqueous phase in which the nZVI particles are suspended. Therefore, our current understanding is that the above chemical reaction takes place within the water phase, and hence nanoremediation is mostly effective downstream of the contamination source.

Although aquifer contamination is a large-scale problem, water flow and contamination entrapment ultimately occur at the pore scale. Study of the pore-scale fluid distributions, therefore, sheds light on the key control mechanisms behind the nanoremediation process and how it can be optimized. Previous four-dimensional (4D) (time-resolved three-dimensional [3D]) experiments, mostly conducted using the X-ray–computed microtomography (µCT) technique, have provided pore-scale observation of processes that control multiphase flow in porous media. Examples include snap-off (24), Haines-jumps events (4, 5), and in situ formation/flow of emulsions (25, 26). Specifically, pore-scale fluid arrangements are shown to change substantially when a liquid-saturated porous medium accommodates a gas phase (27). As the nanoremediation chemical reactions produce gas, it is important to understand whether and how the release of this gas changes the pore-scale fluid arrangements.

## Results

We present the outcomes of a 4D experiment in which we captured a sequence of 3D images of a bead pack (and the contaminants it hosts) during injection of a nZVI suspension. The 3D data, acquired using the µCT technique, are in the form of high-resolution images that allow observation of the internal structure of the sample, nondestructively. Our main hypothesis was that the emergence of a gas phase (product of the chemical reaction) will cause fluid redistribution at the pore scale, changing the fluid arrangements and, hence, impacting the DNAPL entrapment. This redistribution is favorable if it reduces the trapping and allows permanent removal of the DNAPL residual phase. However, it could also pose a risk of contaminating the clean zones of the aquifer by mobilizing the trapped residual DNAPL and is, therefore, worth careful investigation.

**Fig. 2.**
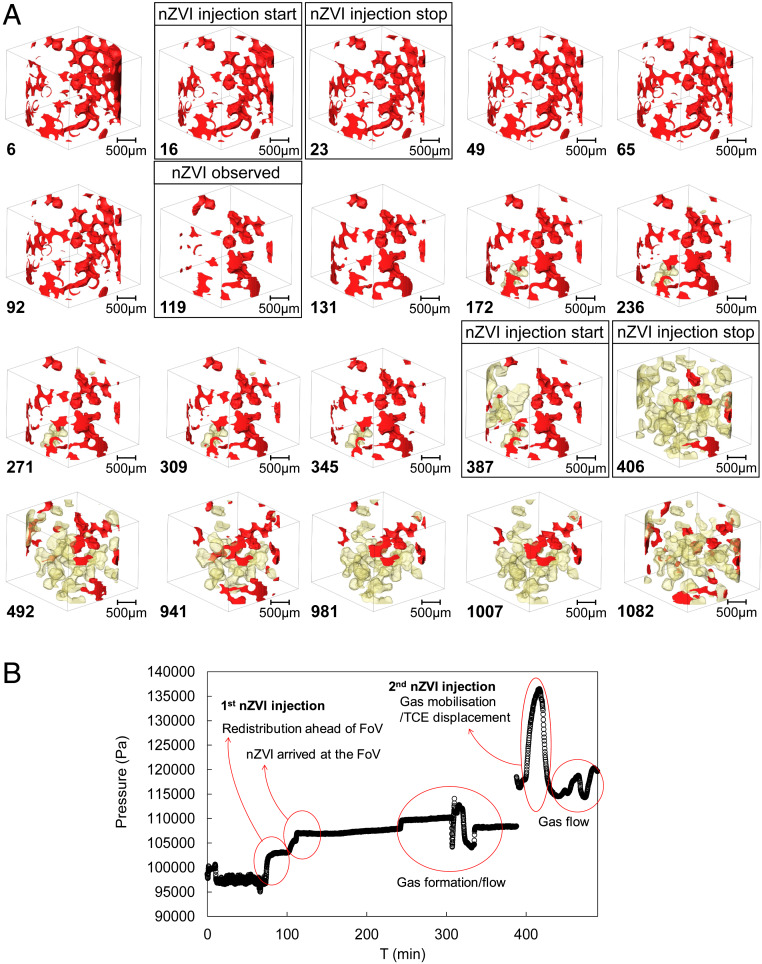
(*A*) Three-dimensional renderings of the TCE phase as a function of time (noted at *Bottom Left* of each image, in minutes). The black boxes show the time steps of specific significance. (Scale bar, 500 µm.) TCE and gas are rendered in red and light yellow, respectively. (*B*) Injection pressure as a function of time.

The glass beads were water wet. The DNAPL phase studied was TCE. The experimental setup is discussed in *Materials and Methods*. The fluid injection sequence and the image acquisition parameters (times, resolution, etc.) are presented in *SI Appendix*, Table S2. The sample was initially fully saturated with water. To eliminate TCE dissolution this water was saturated with TCE (solubility 1.28 g/L). The initial condition of a groundwater contamination source with a residual DNAPL phase was mimicked by injection of pure TCE, followed by water (TCE saturated) injection, which displaced mobile TCE from the pore space. At this stage TCE was imaged to occupy 12.42% of the pore space (T = 6 min in Fig. 2*A*) in the form of disconnected droplets/ganglia.

**Fig. 3.**
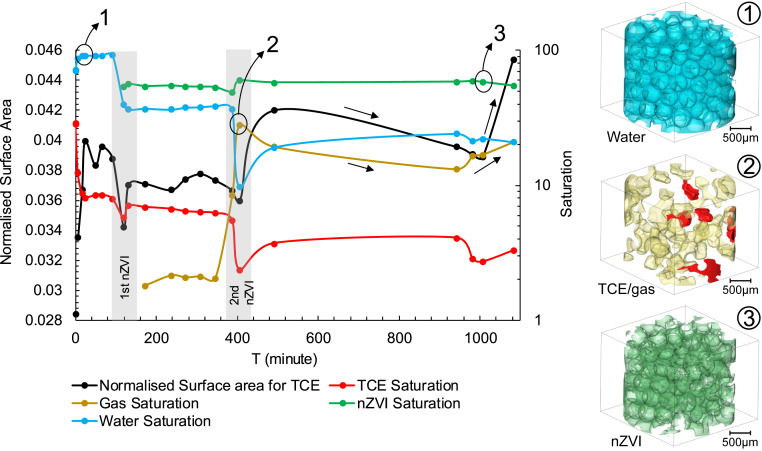
Fluid phase saturation and the normalized surface area for the TCE phase within the imaged section of the bead pack as a function of time. The 3D renderings displayed at *Right* show fluid distributions within the FoV. TCE, red; gas, yellow; water, blue; and nZVI, green.

The nZVI suspension (50 g/L) was subsequently injected in two steps, separated by ∼6 h of no flow, mimicking a two-stage field injection of the reactant. A final no-flow step was also recorded. The first nZVI injection (200 µL/min) started at T = 16 min and finished at T = 23 min. Three-dimensional images were collected during the following 6 h. This nZVI injection reduced the TCE saturation to 7.91% by T = 92 min and yet this was before the nZVI front reaches the imaging field of view (FoV) (Fig. 2*A*). This means that the extra pressure drop, caused by the injection of nZVI suspension, has displaced ∼36% of TCE outside the FoV. The pressure vs. time plot is shown in Fig. 2*B*. It is observed that most of this first TCE removal is from the pores touching the tube boundary. This is due to the fact that TCE preferentially flows along the tube to which it is wetting. Conversely, the TCE phase within the porous medium (away from the boundaries) is not affected by the flow ahead of the nZVI front. The images first display the arrival of the nZVI at the FoV at T = 119 min. Fluid saturations are plotted in Fig. 3. As a result, the TCE saturation is reduced a further 2.17%, making the remaining TCE saturation 5.74%. The arrival of the nZVI at the FoV displays a further increase in pressure as shown in Fig. 2*B*. The following image (T = 131 min) shows that the TCE within the pore space remained trapped while some extra TCE moved back into the FoV by flowing along the column walls.

At T = 172 min the emergence of a gas phase was observed within the FoV; this is shown in yellow color in Fig. 2*A*. The presence of this gas phase has caused some pressure disturbance which was captured by the gauge. The second nZVI injection started at T = 387 min. A clear increase in the injection pressure was recorded. During the second injection, the TCE saturation sharply decreased to 2.34%, the lowest measured in this experiment. This time the reduction of TCE saturation is due to its displacement by the gas phase. Gas saturation increases from 8.51 to 28.05% from T = 387 min to T = 406 min. The presence and flow of such significant amounts of gas are recorded by the gauge as pressure instabilities (Fig. 2*B*). Once again, TCE flows back into the FoV along the tube walls during the no flow period and hence its saturation increases to 3.72% at T = 492 min.

The gas movement from outside the FoV into the imaged section clearly has a significant impact on remobilizing the TCE droplets from the pores they were previously trapped in. The amount of gas is significant; at its highest there is 28.05% of gas imaged within the pore system (T = 406 min). The inlet and outlet valves were closed between T = 492 and T = 941 min; hence pressure was not recorded over this period. The images captured at T = 941 min (next day) show the TCE/gas distribution within the FoV continues to change with the gas saturation dropping to 13.17%, while the TCE saturation increased to 4.08%. Gravity-driven phase segregation is potentially a main player in this continuous redistribution under the no flow condition. A final step involved injection of water, which resulted in further remobilization of both the TCE and gas phases as shown in Fig. 3.

A key factor to investigate here is the change in TCE surface area as a result of the fluid injections. For in situ techniques the degradation extent depends on the contaminant/chemical (or nanoparticle) interface area. Therefore, larger TCE surface areas facilitate its degradation. The reported TCE surface area (Fig. 3) is normalized against the TCE volume to allow direct comparison for the different stages of this experiment. Our data show that the normalized TCE surface area is significantly influenced by the formation and flow of the gas phase. Gas flow into the FoV, at T = 406 min, has resulted in ∼17% increase in the TCE surface area. Subsequent images show that as the gas saturation decreases the TCE surface area decreases too. Finally, the TCE surface displays a sharp increase during the last water injection where the gas saturation is increased too.

The nanoparticles were first suspended in water 1 mo prior to the experiment. As a general rule, the reactivity of particles reduces with time due to corrosion, but is not significantly depleted even in the presence of a thick oxidized surface layer, unless the greater part of nZVI is consumed (28, 29). Therefore, it is important to examine the chemical reactivity of particles prior to the implementation of the nanoremediation process. The reactivity of the particles we used in this experiment was measured to be 46% of fresh particles at the time of performing this experiment.

### Pore-Scale Dynamics of Nanoremediation.

**Fig. 4.**
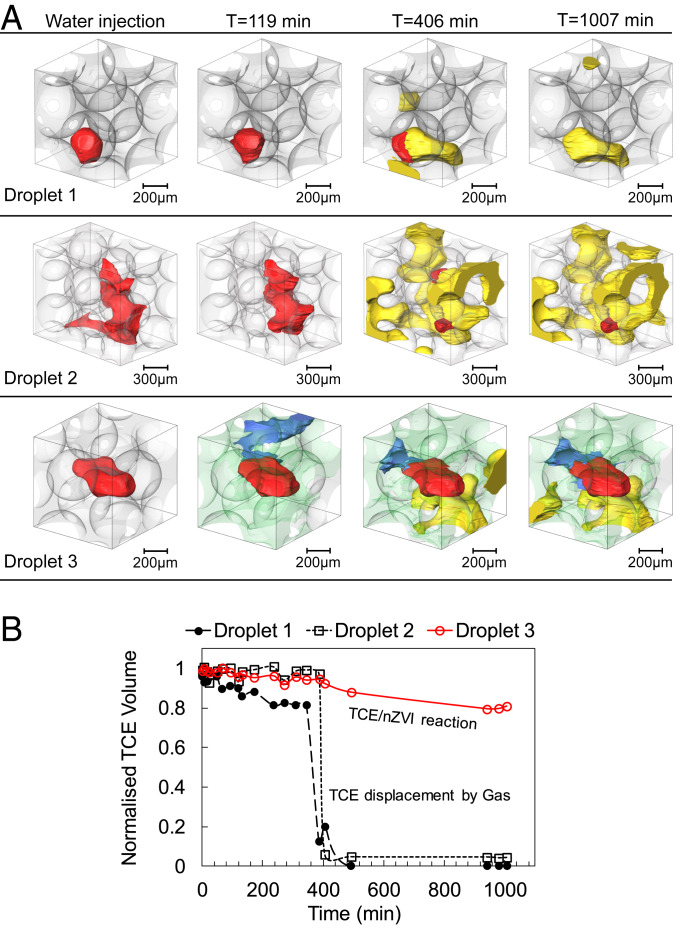
(*A*) Example TCE droplets (red) rendered at different time steps. Droplets 1 and 2 are remobilized by the gas phase (yellow). Droplet 3 shows gradual degradation of TCE and formation of water film (blue). nZVI is rendered in green. (*B*) Normalized TCE volume as a function of time for the three droplets shown in *A*.

**Fig. 5.**
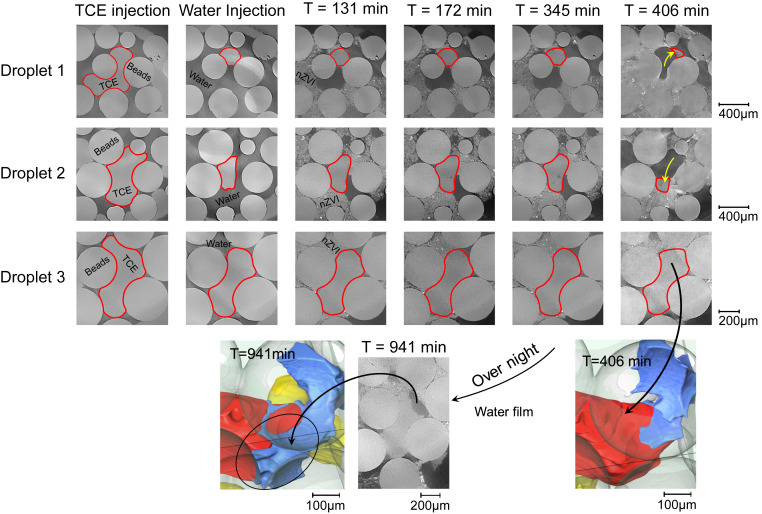
Example 2D µCT slices through the droplets shown in Fig. 4. The round objects are the beads. Shown are TCE (light gray, homogeneous), water (medium gray), gas (dark gray, homogeneous), and nZVI suspension (irregular light gray with bright spots). TCE phase boundaries are highlighted with red lines for better clarity. Gas displacing TCE is shown with yellow arrows. For 3D renderings colors are explained in the Fig. 4 legend.

At the pore scale the significance of gas formation in redistributing fluids is evident. Fig. 4*A* (droplets 1 and 2) provides examples of such fluid rearrangements as a result of gas movement into pores previously occupied by TCE. As two nonwetting phases both TCE and gas prefer to occupy the center of the pores, i.e., pore bodies. Water and nZVI, both acting as a wetting phase, spontaneously imbibe into pore throats and can form wetting films and/or corner films. It is also worth mentioning that the nZVI suspension does not readily mix with water at first contact. Images shown in Fig. 5 display a clear interface between the nZVI suspension and the water phase.

TCE surface tension (28.7 mN/m) is smaller than water surface tension (72 mN/m) and TCE/water IFT (39.6 mN/m) (30). This means that ∼62% less energy is required to create a unit of gas/TCE contact interface compared to the gas/water interface. As a result, it is expected that the gas/TCE interface will preferentially form, where possible, to minimize the energy of the system.

Fig. 4*A* (droplets 1 and 2) shows examples where trapped TCE droplets were displaced out of the pores confining them by the emerged gas phase. The 3D renderings are created to show these individual droplets at four different time steps of T = 0 (after water injection), T = 119 min (where the nZVI was first observed in the FoV), T = 406 min (at the end of the second nZVI injection), and T = 1,007 min (the next day). The first two time steps show that the TCE remains as a residual phase trapped within a single pore or multiple neighboring pores. From the images it is evident that the nZVI phase does not directly displace the trapped TCE droplets. It is rather the gas flow that remobilizes the TCE at T = 406 min. Fig. 4*B* shows the TCE volume imaged within this cropped box at different time steps. The volumes are normalized against the initial TCE volume at T = 0 to allow direct comparison. The sudden drop in normalized TCE volume for droplets 1 and 2 shows the effectiveness of gas displacement in removing TCE droplets.

Droplet 3 is not displaced by the gas phase after the second nZVI injection (T = 406 min). In contrast, our images show that the TCE has gradually degraded at the TCE/nZVI interface in a slower process. In Fig. 4*B* the extent of this degradation process is shown in terms of the decrease in TCE volume; here ∼13% of the TCE is degraded after 1 d. The images also capture the presence of a water film which appears to be sandwiched between the nZVI and TCE phases. This is better shown in Fig. 5 using the µCT slices. This evidence shows direct degradation of a chlorinated hydrocarbon residual phase as it comes in contact with nZVI within porous media. Our previous understanding has been that only the dissolved TCE reacts with nZVI, which is mainly the reason behind nZVI injection downstream of the contamination source (i.e., within the contamination plume) and not directly targeting the source zone.

### Impact of Gas Formation and Flow on Water Permeability.

Natural groundwater flow can result in transport of dissolved contaminants into clean areas of the groundwater system. Controlling the flow of contaminated water can therefore contribute to limiting the spread of dissolved contaminants. Within the context of groundwater systems the aqueous phase is commonly the wetting phase, while the chlorinated solvents and any gas phase act as nonwetting phases. For the wetting aqueous phase, at the pore scale, the flow happens both in bulk (for pores solely occupied by the water phase) and through film/corner flow (for pores with pore bodies occupied by a nonwetting phase). Corner/film flow is significantly slower than bulk flow and hence introduction of a nonwetting phase that could preferentially occupy pore bodies significantly reduces the flow (spread) of the contaminated water (31).

**Fig. 6.**
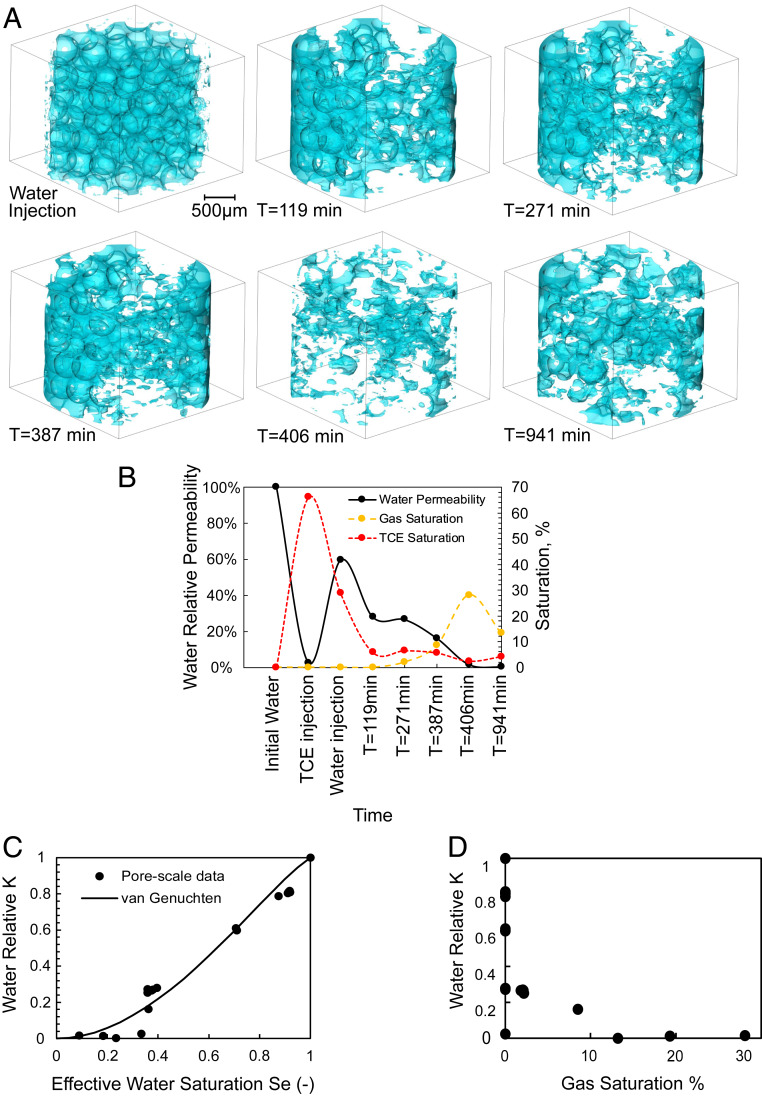
(*A*) The 3D rendering of the water phase at different time steps. (*B*) Water relative permeability as a function of time. (*C*) Water relative permeability calculation based on 3D images shown in *A*. (*D*) Water relative permeability as a function of gas saturation.

From this experiment, effective permeability of water is calculated by solving the Navier–Stokes equation on 3D binary images of the water phase at each time step. This means at each step, only the segmented image of the water phase is considered in these calculations (single phase). It is clear that multiphase flow simulations capable of accounting for effects such as viscous coupling can provide a more accurate picture of this process. Fig. 6*A* shows 3D renderings of the water phase at different time steps. The absolute permeability is calculated based on the 3D image of the entire (imaged) pore space. Water relative permeability is calculated by normalizing its effective permeability against the absolute permeability of the sample (Fig. 6 *B*–*D*).

Fig. 6*B* shows that the measured water relative permeability decreases from ∼60% to ∼26% as a result of the first nZVI injection (initially immiscible with water) and further reduces to less than 1% as a result of gas formation and flow into the FoV. This shows substantial reduction of contaminant spreading is achieved during application of nanoremediation technology.

We use the soil–water characteristic curve model of van Genuchten (32) to model water permeability at the Darcy scale. This model describes how fluid permeability (*K*) correlates with its saturation, as shown in Eq. 1,

$$K\left(S_{e}\right)=K_{\text{sat}}S_{e}^{1/2}{\left[1-{\left(1-S_{e}^{1/m}\right)}^{m}\right]}^{2},$$ [1]

where m is a fitting parameter, $K_{\text{sat}}$ is the absolute (saturated) permeability [L^2^], and $S_{e}$ is the effective saturation [-], defined as

$$S_{e}=\frac{\theta -\theta_{r}}{\varphi -\theta_{r}}$$ [2]

in which φ is the total porosity of the medium, θ the water content, and $\theta_{r}$ the residual water content or irreducible moisture content (in our case, assumed equal to 3%, typical for bead packs) (33). m (known as the sorting coefficient) is related to the pore size distribution of the porous media. For wider pore size distributions larger values of m are expected (34). Here, for m equal to 1.3 a good agreement between the pore-scale and Darcy-scale K vs. S trends is achieved (*R*^2^ = 0.95) (Fig. 6*C*). The match between the pore-scale data and the van Genuchten (32) model suggests that the processes observed in the packed column are in line with the behavior expected at the Darcy scale, even though the size of the imaged sample is relatively small.

## Materials and Methods

The experimental setup is shown in *SI Appendix*, Fig. S1. The porous medium was a glass bead pack (internal diameter = 2.5 mm, length = 33 mm, φ = 38%). Absolute permeability is estimated to be K = 1.7 × 10^−11^ m^2^ using the Kozeny–Carman (KC) equation. The 3D images were used to measure the geometric properties of this porous medium (e.g., specific surface area and pore-space tortuosity) (*SI Appendix*, Table S1). The bead sizes range from 250 to 500 µm to represent medium to coarse sand. See *SI Appendix*, Fig. S2 for the bead/pore size distributions. Contaminated aquifer systems commonly display porosity, permeability, and pore sizes similar to this sample.

The beads were acid washed prior to the experiments to remove any metal oxide impurities from the bead surfaces. The nZVI is NanoFer 25S acquired from NANOIRON in the form of a concentrated aqueous suspension which was further diluted (18.2 MΩ.m deionized water) to 50 g/L concentration. The nZVI particles have an organic and biodegradable coating which enhances the particle suspension. The reactivity of these particles was examined prior to the flow experiments using 1) measurement of the evolved gas volume during nZVI reaction with sulphuric acid and 2) X-ray photoelectron spectroscopy analysis (data presented in *SI Appendix*, Fig. S3). The TCE was doped with iododecane (75/25 vol%) to enhance the fluid contrast on the captured µCT images.

The flow cell was designed and manufactured at the Brazilian Synchrotron Light Laboratory (LNLS). It is made of two stainless steel end pieces and a polytetrafluoroethylene (PTFE) tube (main body) in which the glass beads were packed. A back-pressure regulator of 138 kPa was attached to the cell outlet in the start of the experiment to ensure there are no air bubbles trapped within the system. This regulator was removed later after the system was bubble-free. A pressure gauge recorded the injection pressure throughout the experiment. All injections were performed from bottom to top.

The experiment started from initial water (saturated by TCE) injection in the system followed by injection of the TCE phase (q = 50 µL/min, N_c_ = 6.15 × 10^−6^, V = 38.62 m/d). Subsequently, we injected TCE-saturated water (q = 10 µL/min, N_c_ = 1.23 × 10^−6^, V = 7.72 m/d) to remove the mobile TCE and establish an initial point where the TCE droplets are trapped and are no longer mobile. The injections were performed within the capillary-dominated flow regime (threshold N_c_ is 10^−5^ to 10^−6^) (35–37). The nZVI suspension was then injected (q = 200 µL/min, N_c_ = 2.46 × 10^−5^, V = 154.48 m/d), while a series of 3D images were collected. The nZVI injection was performed at a higher flow rate to overcome injection challenges which are also known at the field scale. The nZVI concentration was kept constant at levels practiced in field injections (50 g/L). The experiments followed the steps outlined in *SI Appendix*, Table S3.

The 3D images were collected during and after fluid injections. Each 3D scan is composed of 200 projections over a 180° rotation, with an exposure time of 1.7 s, leading to a scan time of ∼6 min. Reconstructed images were obtained by an in-house filtered back projection-based algorithm (38). The 3D images were segmented into binary images representing the water, TCE, nZVI, and gas phases. Details of the image processing steps are described in *SI Appendix* and the workflow is presented in *SI Appendix*, Fig. S4. In brief, reconstructed images were filtered using the nonlocal means filter prior to segmentation. Image segmentation was performed using a combination of Waikato Environment for Knowledge Analysis (WEKA) (39) segmentation, watershed (40) segmentation, and thresholding (41), followed by manual corrections where needed. The WEKA algorithm was used to segment the beads and the nZVI phases, since their distinct texture allowed reliable segmentation using the machine-learning approach. For the TCE, water, and gas phases watershed segmentation and thresholding were applied. After segmentation, connected objects on the binary images were labeled (42) to perform quantitative measurements (e.g., volume, area, position) for each fluid phase. The beads were removed (masked) to assist with image segmentation (*SI Appendix*, Fig. S5).

The dimension of the collected images is 1,024 × 1,024 × 1,024 voxels, which is 3.36 × 3.36 × 3.36 mm^3^. The image voxel side length is 3.28 µm. Quantitative data and 3D renderings used in this paper were extracted from a smaller field of view of 1,024 × 1,024 × 624 (3.36 × 3.36 × 2.05 mm^3^) due to artifacts (typical) observed at both ends of the image (*SI Appendix*, Fig. S6). Corrected image resolutions are calculated using the Fourier ring correlation approach (43) and are reported and discussed in *SI Appendix*, Table S2. In short, the image resolutions range from 10.9 µm to 27.54 µm with a median of 16.56 µm. The experiment was performed at the X-ray microtomography beamline at the Brazilian Synchrotron Light Laboratory, Brazilian Center for Energy Research and Materials (LNLS/CNPEM), using a polychromatic beam, filtered by a Si filter. The X-ray energy profile available at this beamline is shown in *SI Appendix*, Fig. S7.

Water relative permeability was numerically estimated by solving the Navier–Stokes equation, considering an incompressible fluid and steady-state flow, followed by applying Darcy’s law to obtain the permeability coefficients. This calculation was performed on the 3D images of the connected fluid/pore space phase using the permeability computation module from the Avizo (44) software.

The dataset generated in this study has been deposited in the Figshare repository and can be accessed at https://doi.org/10.6084/m9.figshare.12053607.v1 (45).

## Conclusions

While the nanoremediation concept is proved to be successful at laboratory, pilot, and field scales, the existing practice is far from optimized, particularly when applied close to the secondary source of contamination, where residual DNAPL is still present as a segregated phase. This contribution provides insights into the pore-scale physics of groundwater remediation using nanotechnology. Specifically, our study presents evidence on the dynamics of the in situ remediation of groundwater contaminated with TCE as a result of nZVI injection. This study is focused on source zone remediation by nZVI, which is not the current common practice.

Our data show that the two main mechanisms that drive the nanoremediation process, at the pore scale, are 1) formation and flow of a gas phase causing remobilization of the trapped TCE (residual) phase and 2) direct degradation of the TCE phase at the TCE/nZVI interface. The series of images collected during this experiment enables us to directly monitor the dynamics of the nanoremediation process within the field of view and beyond. The gas-induced TCE remobilization causes rapid change in the in situ TCE distribution in the host sediment. Within the imaged section of our sample TCE showed a ∼57% reduction in saturation as a result of gas-induced remobilization, which is substantial. The direct TCE degradation at the TCE/nZVI interface was observed only in the images captured on the following day after the nZVI injection. This shows that the direct degradation is a more gradual process. While gas formation/flow can occur in hours, the direct degradation will require days to weeks. As an example, the TCE droplet presented in this paper showed a ∼13% reduction in volume as a result of direct degradation by nZVI. A thick water film sandwiched between the TCE and the nZVI phase evidences this direct degradation.

Gas formation is a well-known effect in field application of remediation technologies. While it is commonly considered as an evidence for the effectiveness of the applied process, it causes a range of challenges/risks; e.g., exposure to the released gas has health and safety implications for residents and workers in contaminated sites. Within this contribution we focus on closing the knowledge gap between pore/Darcy scales, specifically by studying the impact of gas formation on mobility of DNAPLs and spread of contaminants. Our findings suggest that gas-induced DNAPL remobilization can displace the previously trapped DNAPL phase. On the other hand, the calculated relative permeability for the aqueous phase displays significant reduction as a result of gas formation and flow. The pore-scale preference of gas for occupation of pore centers derives from this significant reduction in water permeability. Both of these findings open perspectives for potential in situ application of nanoremediation for direct treatment of DNAPL sources containing a residual phase. It also shows application of any remediation process that produces a substantial amount of gas can limit further spreading of the contaminated water (dissolved DNAPL) into clean parts of the groundwater system, although temporarily.

Based on our experimental observations, gas formation is expected to occur relatively shortly after nZVI injection. It can result in a partial and fast remobilization of the residual DNAPL phase. Such remobilization will be limited to the source area, due to the concurrent permeability reduction. In contrast, nZVI chemically reduces the contaminant (dissolved fraction) over a longer term. While field-scale validation is needed to further confirm these observations, our pore-scale study demonstrates that there is value in developing a combined nanoremediation/soil flushing approach for residual DNAPL treatment. Such a combined process can speed up source depletion and consequently improve the overall efficiency of the remediation process.

## Supplementary Material

Supplementary File
