# Supplementary material for: Pore-scale investigation of the use of reactive nanoparticles for in situ remediation of contaminated groundwater source
Source: Proc Natl Acad Sci U S A. 2020 Jun 2;117(24):13366–73. doi: 10.1073/pnas.1918683117 (PMC7306790; doi:10.1073/pnas.1918683117)
Supplement: Supplementary File [file pnas.1918683117.sapp.pdf]

Supplementary Information

Title: Pore scale Investigation of the Use of Reactive Nanoparticles for In situ Remediation of Contaminated Groundwater Source

Authors List: Tannaz Pak[1], Luiz Fernando de Lima Luz Jr[2], Tiziana Tosco[3], Gabriel Schubert Ruiz Costa[4], Paola Rodrigues Rangel Rosa[4], and Nathaly Lopes Archilha[4]
[1]Teesside University, [2]Federal University of Parana, [3]Polytechnic University of Turin, [4]Brazilian Synchrotron Light Laboratory (LNLS), Brazilian Center for Research in Energy and Materials (CNPEM), Zip Code 13083-970, Campinas, Sao Paulo, Brazil.

www.pnas.org/cgi/doi/10.1073/pnas.1918683117

1- Experimental Set-up

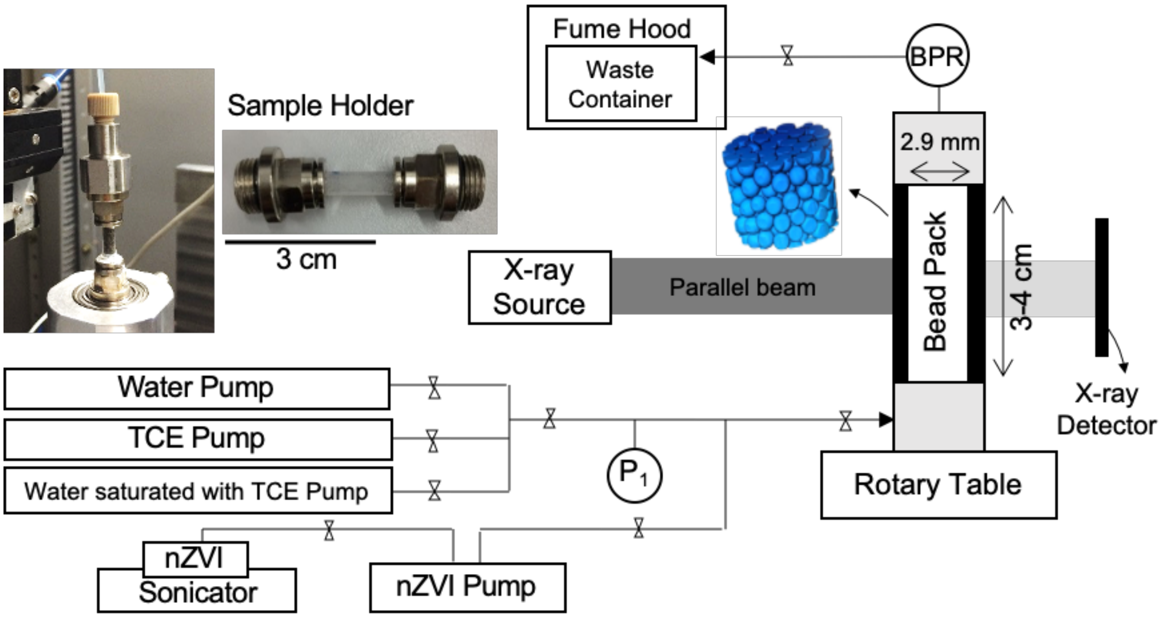

Fig. S1: The experimental set-up used in this work.

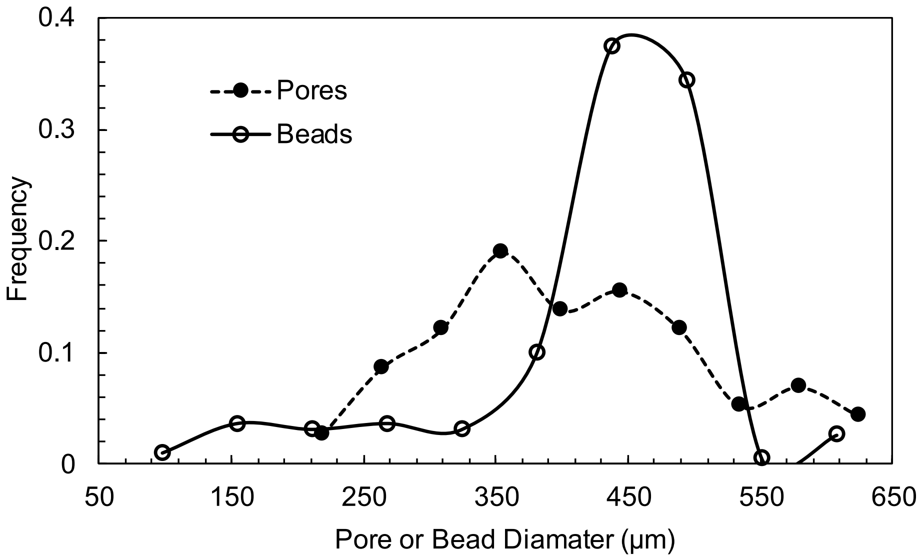

Fig. S2: Bead/pore size distribution for the column used in this experiment

2- X-ray photoelectron spectroscopy (XPS) results for nZVI

nZVI degrades with time. We performed X-ray photoelectron spectroscopy (XPS) analysis on a sample of our nZVI at the time of experiments for which the results are presented in this work. This was to ensure the nZVI is reactive. Fig. S3 shows the results of this XPS analysis. It is clear that significant amount of Fe^0 exists in the sample at this point.

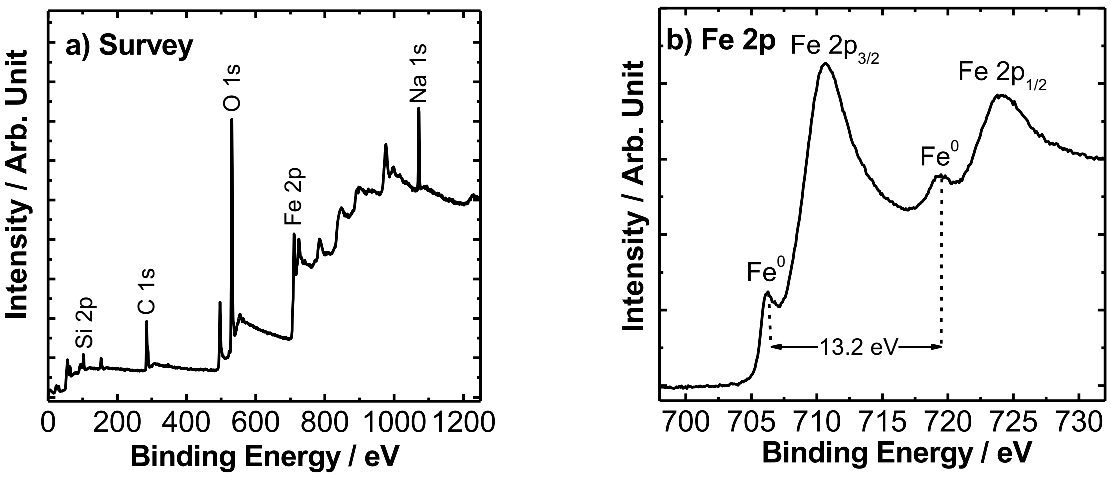

Fig. S3: XPS spectra of a sample of our nZVI, including wide-scan or survey (a) and high resolution spectrum (b) measured in bonding energy range of Fe 2p signal.

3- Permeability calculation

The calculated permeability using Kozeny-Carman (KC) equation is discussed here. The 3D images collected in this experiment enable us to directly measure the geometric properties of this porous media. To achieve this, we use the image of the bead pack collected at the start of the experiment (i.e. initial water saturation step). The *3D Area/3D Volume* for this sample is measured to be equal to 3.86×10^4 1/m. Considering the glass density of 2400 kg/m^3 we arrive at the specific surface area of 16.09 m^2/kg for this sample. We also measure the pore-space tortuosity to be 1.5. The KC permeability is calculated using Eq 1 to be 1.7×10^{-11} m^2 (i.e. 17.23 D).

$$k = \frac{\varphi^3}{c\tau^2 A^2 (1-\varphi)^2} \qquad \text{(Eq1)}$$

Table S1: Parameters used to calculate permeability based on Kozeny-Carman Eq.

k, permeability, D	17.23
k, permeability, m^2	1.72×10^{-11}
φ, porosity	0.38
c coefficient	2.5
Area per unit volume, m^2/m^3	3.86×10^4
A, Specific surface area, m^2/kg	16.09
τ, L_t/L_0	1.5

4- Flow Experiment Steps

Table S2: Image resolutions calculated using the FRC approach. Pixel size 3.28 µm.

Step	Start Time	Finish Time	Acquisition time	Resolution (voxels)	Resolution (µm)	Number of projections
Initial Water Inj	-------	-------	-------	2.974	9.75	2000
TCE Inj	4:23:10 PM	5:11:00 PM	00:47:50	6.789	22.27	2000
Water Inj	6:13:48 PM	7:29:45 PM	01:15:57	8.396	27.54	2000
T02	8:37:22 PM	8:42:40 PM	00:05:18	8.017	26.3	200
T03	8:47:39 PM	8:52:58 PM	00:05:19	6.501	21.32	200
T04	8:53:30 PM	8:58:53 PM	00:05:23	6.546	21.47	200
T05	9:00:52 PM	9:25:35 PM	00:24:43	6.333	20.77	1000
T06	9:28:23 PM	9:36:10 PM	00:07:47	6.217	20.39	300
T07	9:42:46 PM	10:09:08 PM	00:26:22	5.09	16.7	1000
T08	10:10:01 PM	10:18:15 PM	00:08:14	4.123	13.52	300
T09	10:27:35 PM	10:35:54 PM	00:08:19	7.676	25.18	300
T10	10:39:06 PM	11:06:41 PM	00:27:35	4.774	15.66	1000
T11	11:19:43 PM	11:47:38 PM	00:27:55	3.983	13.06	1000
T13	12:23:52 AM	12:54:13 AM	00:30:21	3.899	12.79	1000
T14	12:58:34 AM	1:29:55 AM	00:31:21	3.938	12.92	1000
T15	1:36:42 AM	2:08:34 AM	00:31:52	3.988	13.08	1000
T16	2:13:01 AM	2:45:56 AM	00:32:55	3.959	12.99	1000
T17	2:55:13 AM	3:05:32 AM	00:10:19	5.917	19.41	300
T18	3:17:02 AM	3:51:55 AM	00:34:53	4.23	13.87	1000
T20	4:42:12 AM	5:56:24 AM	01:14:12	3.322	10.9	2000
T21	12:11:08 PM	12:38:35 PM	00:27:27	3.89	12.76	1000
T22	1:04:38 PM	1:13:25 PM	00:08:47	6.25	20.5	300
T23	1:15:04 PM	1:24:23 PM	00:09:19	5.743	18.84	300
T28	2:29:25 PM	2:58:52 PM	00:29:27	5.008	16.43	1000
T29	1:50:39 PM	2:32:46 PM	00:42:07	4.048	13.28	2000

5- Image Collection, Processing and Quantification

As shown in Fig. S4 reconstructed images were segmented using a combination of available segmentation algorithms, implemented in ImageJ and Avizo. Segmentation of the glass beads (in initially water-saturated image) and nZVI phases were performed using the WEKA segmentation algorithm via FIJI (which is a distribution of ImageJ). The used Trainable Weka Segmentation[1] (TWS) plugin works based on machine learning and is hence an iterative process of training and segmenting until an acceptable result is achieved. The TWS showed better results taking raw images as input. For other phases (i.e. TCE, water, and gas) we used algorithms available in Avizo. First, the volume edit module was used to separate the sample from its

background. Next, images were filtered using the non-local means[2] filter followed by the unsharp mask[3] filter within Avizo.

To facilitate fluid phase segmentations, the glass beads (obtained using TWS) were removed from the grayscale image by masking. The remaining fluid phases (i.e. TCE, gas, nZVI, and water) were segmented using a combination of watershed and thresholding, followed by manual corrections where needed. The presented quantifications were performed using the label analysis module within Avizo. Permeability was estimated by taking the water label as input, using the Absolute Permeability Experiment Simulation module within Avizo.

The radiographs were collected using the filtered (Si filters, 900μm) radiation produced by the 1.67T bending magnet of the 1.37 GeV storage ring. The polychromatic (pink) beam has an energy peak at ~15.2 keV (~50 % bandwidth), see Fig. S7. The detector system is composed by a LuAg:Ce scintillator, a 5X objective lens, which magnifies and focus the image in a CCD sensor (PCO.2000).

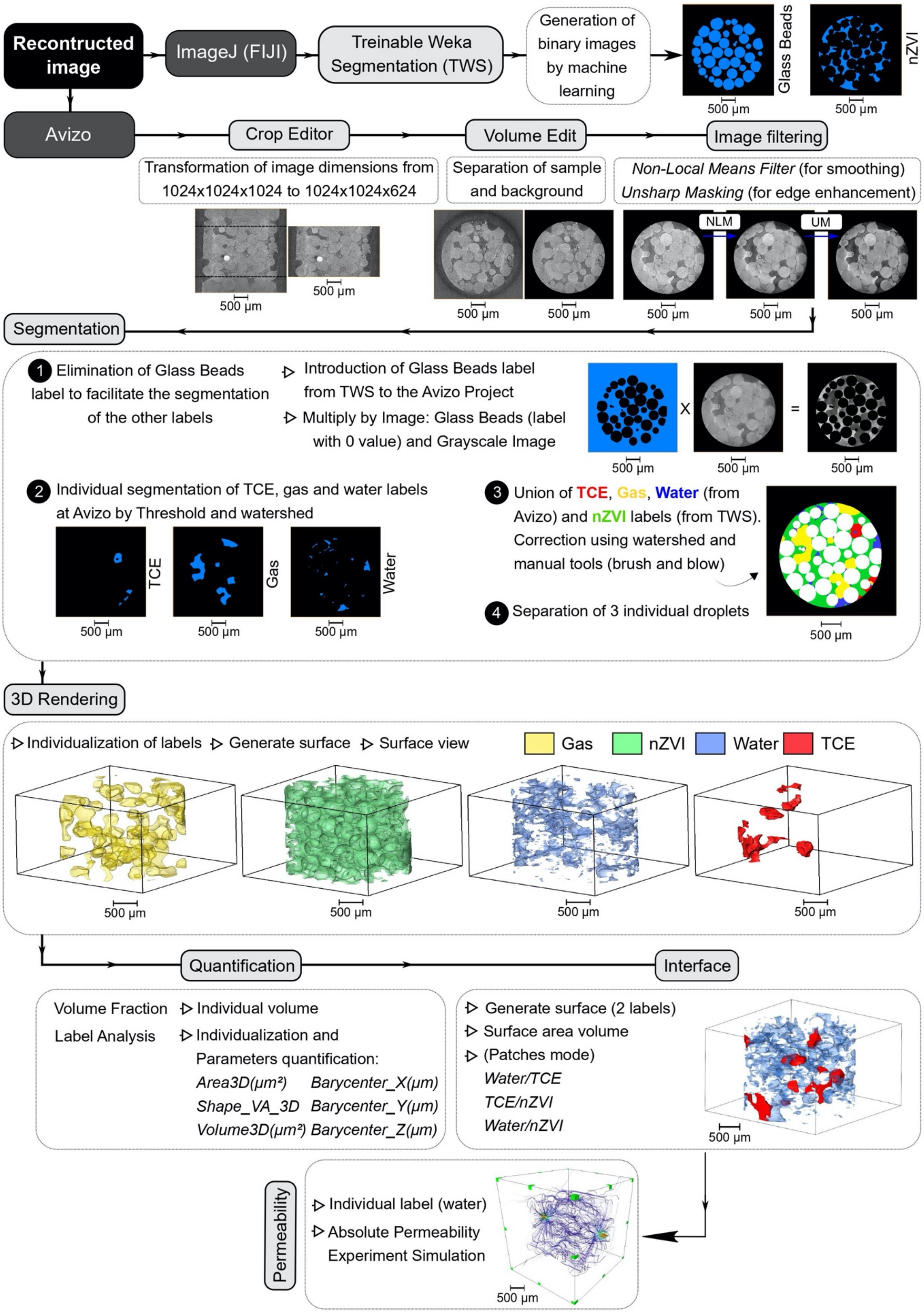

Fig. S4: Image processing and quantification workflow

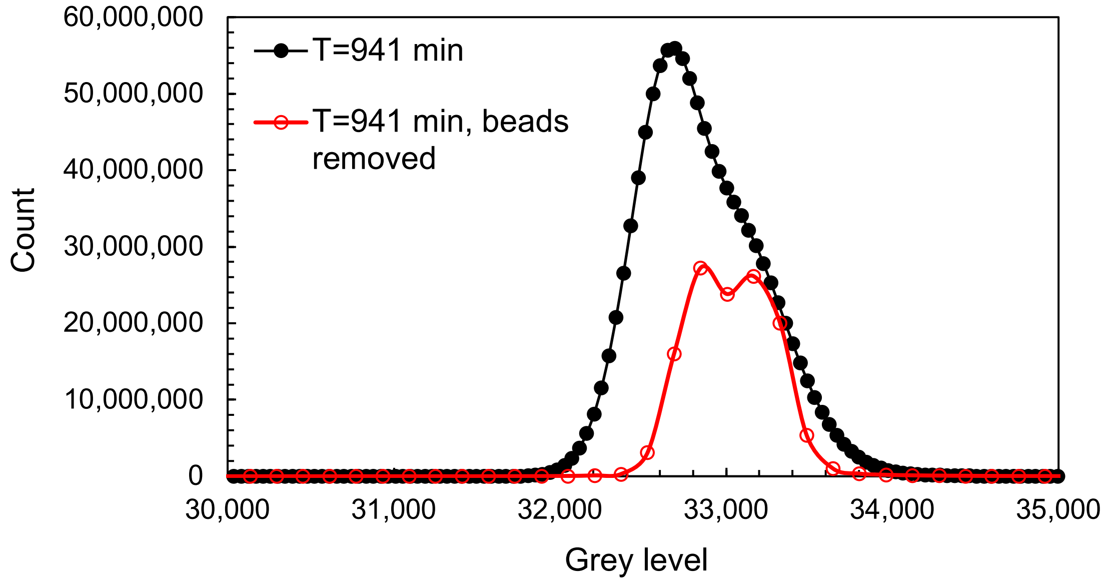

Fig. S5: Image histogram plotted for the image captured at T = 941 min.

Fig. S6 shows common image artefacts that were observed at both ends of the reconstructed images. In order to eliminate those artefacts for data analysis the images were cropped to a smaller field of view of 1024×1024×624 (3.36×3.36×2.05 mm^3).

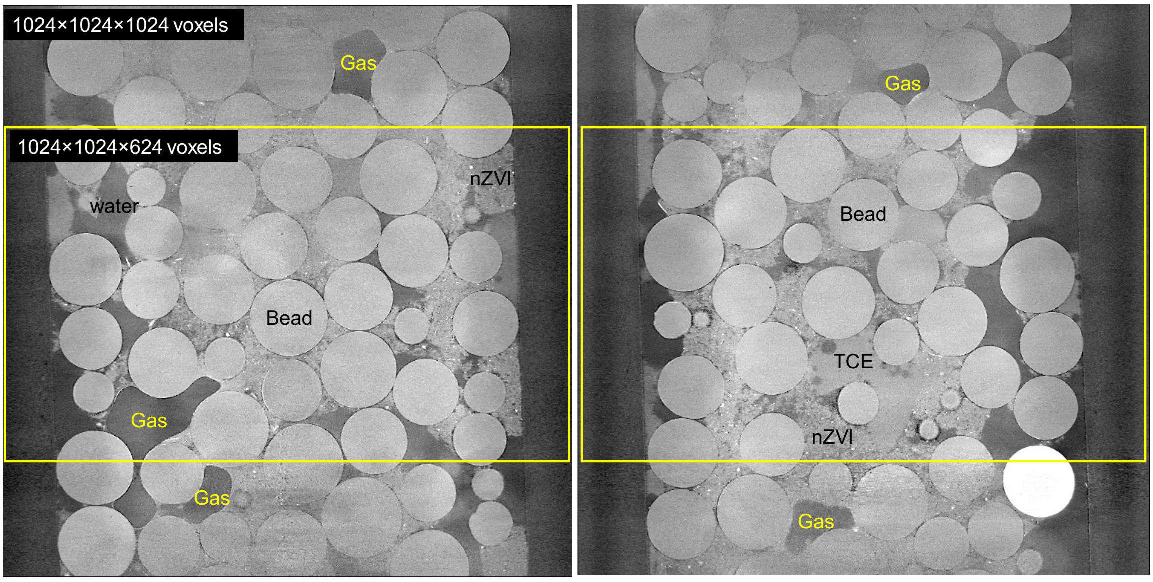

Fig. S6: Example μCT slices of the beakpack at T=271 min. It is clear that there is gas phase outside the field of view selected here (shown by the yellow box) for the data quantification and 3D renderings. Outside this box the images are less sharp due to end effects.

Corrected image resolutions is calculated by applying the Fourier Ring Correlation (FRC) approach, using the imageJ plugin: Fourier Ring Correlation Plugin. This code takes as input two consecutive reconstructed 2D slices of the 3D image. It evaluates similarities between those two slices in the frequency space to determine the spatial frequency (or resolution threshold) at which both images are consistent. This threshold is directly related to the image resolution.

Table S3: The fluid saturations calculated after each injection step.

Injection Steps	Time step, min	Flow rate (μL/min)	nZVI Saturation, %	TCE Saturation, %	Water Saturation, %	Gas Saturation, %
Initial Water Inj	0	100-1000	0	0	100	0
TCE	0	50	0	66.00	34.00	0
Water Inj	0	10	0	28.68	71.32	0
T02	0	0	0	29.05	70.95	0
T03	6	0	0	12.42	87.58	0
T04	16	Start, 200	0	8.78	91.22	0
T05	23	Stop	0	8.09	91.91	0
T06	49	0	0	8.43	91.57	0
T07	65	0	0	8.51	91.49	0
T08	92	0	0	7.91	92.09	0
T09	119	0	54.16	5.74	40.10	0
T10	131	0	56.39	7.05	36.48	0
T11	172	0	54.30	6.90	37.00	1.80
T13	236	0	54.71	6.68	36.47	2.14
T14	271	0	53.86	6.42	37.66	2.07
T15	309	0	53.58	6.32	38.00	2.10
T16	345	0	53.21	6.21	38.55	2.03
T17	387	Start, 200	49.21	5.53	36.75	8.51
T18	406	Stop	59.89	2.34	9.72	28.05
T20	492	0	57.88	3.72	19.12	19.28
T21	941	0	58.56	4.08	24.18	13.17
T22	981	0	59.32	2.85	21.27	16.57
T23	1007	0	58.17	2.71	22.20	16.92
T28	1082	0	54.69	3.27	20.96	20.91

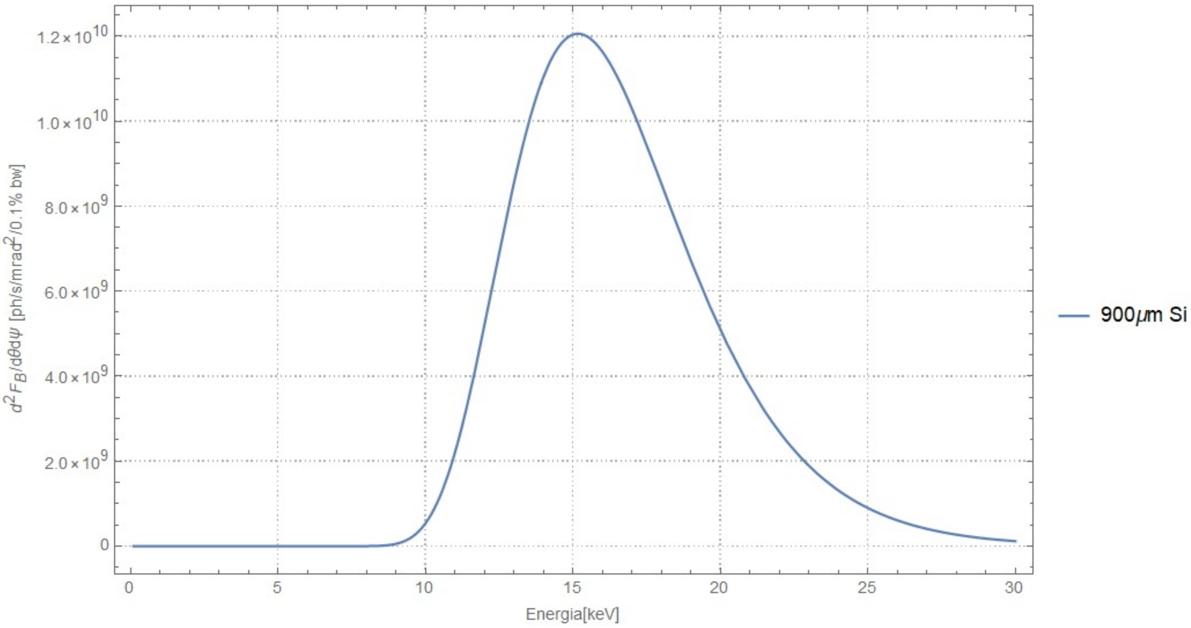

Fig. S7: The polychromatic (pink) X-ray beam at IMX, energy peak at ~ 15.2 keV.

References:

1. Arganda-Carreras, I. *et al.* Trainable Weka Segmentation: a machine learning tool for microscopy pixel classification. *Bioinformatics* **33**, 2424–2426 (2017).

2. Buades, A. ., Coll, B. . & Morel, J.-M. A Non-Local Algorithm for Image Denoising. in *IEEE Computer Society Conference on Computer Vision and Pattern Recognition (CVPR'05)* **2**, 60–65 (IEEE, 2005).

3. Ramponi, G. Warped distance for space-variant linear image interpolation. *IEEE Trans. Image Process.* **8**, 629–639 (1999).
